# Growth factor and pro-inflammatory cytokine contents in platelet-rich plasma (PRP), plasma rich in growth factors (PRGF), advanced platelet-rich fibrin (A-PRF), and concentrated growth factors (CGF)

**DOI:** 10.1186/s40729-016-0052-4

**Published:** 2016-08-22

**Authors:** Hideo Masuki, Toshimitsu Okudera, Taisuke Watanebe, Masashi Suzuki, Kazuhiko Nishiyama, Hajime Okudera, Koh Nakata, Kohya Uematsu, Chen-Yao Su, Tomoyuki Kawase

**Affiliations:** 1 Tokyo Plastic Dental Society, Kita-ku, Tokyo, Japan; 2 Bioscience Medical Research Center, Niigata University Medical and Dental Hospital, Niigata, Japan; 3 Department of Dentistry, National Yang-Ming University, Taipei, Taiwan; 4 Division of Oral Bioengineering, Institute of Medicine and Dentistry, Niigata University, Niigata, Japan

**Keywords:** Growth factor, Platelet-rich plasma, Platelet-rich fibrin, Plasma rich in growth factors, Concentrated growth factors

## Abstract

**Background:**

The development of platelet-rich fibrin (PRF) drastically simplified the preparation procedure of platelet-concentrated biomaterials, such as platelet-rich plasma (PRP), and facilitated their clinical application. PRF’s clinical effectiveness has often been demonstrated in pre-clinical and clinical studies; however, it is still controversial whether growth factors are significantly concentrated in PRF preparations to facilitate wound healing and tissue regeneration. To address this matter, we performed a comparative study of growth factor contents in PRP and its derivatives, such as advanced PRF (A-PRF) and concentrated growth factors (CGF).

**Methods:**

PRP and its derivatives were prepared from the same peripheral blood samples collected from healthy donors. A-PRF and CGF preparations were homogenized and centrifuged to produce extracts. Platelet and white blood cell counts in A-PRF and CGF preparations were determined by subtracting those counts in red blood cell fractions, supernatant acellular serum fractions, and A-PRF/CGF exudate fractions from those counts of whole blood samples. Concentrations of growth factors (TGF-β1, PDGF-BB, VEGF) and pro-inflammatory cytokines (IL-1β, IL-6) were determined using ELISA kits.

**Results:**

Compared to PRP preparations, both A-PRF and CGF extracts contained compatible or higher levels of platelets and platelet-derived growth factors. In a cell proliferation assay, both A-PRF and CGF extracts significantly stimulated the proliferation of human periosteal cells without significant reduction at higher doses.

**Conclusions:**

These data clearly demonstrate that both A-PRF and CGF preparations contain significant amounts of growth factors capable of stimulating periosteal cell proliferation, suggesting that A-PRF and CGF preparations function not only as a scaffolding material but also as a reservoir to deliver certain growth factors at the site of application.

## Background

Platelet-rich plasma (PRP) was originally demonstrated to be effective in the operation of alveolar ridge augmentation and immediately spread to the fields of periodontal and oral maxillofacial surgery [1]. This clinical application was endorsed by evidence that several major growth factors are contained at high levels in PRP preparations. However, for some reasons, such as low handling efficiency, addition of animal-derived thrombin for clotting, and fundamental individual differences, it has been indicated that it is difficult to reproducibly control the quality of PRP preparations at similar levels [1]. To overcome these drawbacks, Anitua developed plasma rich in growth factors (PRGF) by modifying the procedure of PRP preparation [2]. It simplified the preparation protocol and replaced animal-derived thrombin with calcium for clotting.

Platelet-rich fibrin (PRF), a self-clotted preparation of PRP derivative, also overcame these matters. Blood samples collected in the absence of anticoagulants are immediately centrifuged to form fibrin clots. This simple preparation procedure has been widely accepted in various medical fields and spread worldwide. Choukroun, a developer of PRF, further modified it to an advanced form (A-PRF), which is expected to contain a relatively greater number of white blood cells (WBC) [3]. Because of low-speed centrifugation, this fibrin clot is softer than that of the original PRF. On the other hand, concentrated growth factors (CGF), another modified form of PRF, are prepared by repeatedly switching the centrifugation speed and are characterized as a relatively stiffer fibrin clot [4]. Therefore, it has been anticipated that the difference in mechanical characteristics may produce a difference in the growth factor content.

The aim of this study was to address the question as to whether growth factors are equally or more concentrated in A-PRF or CGF preparations and whether these preparations function like a reservoir of major platelet-derived growth factors as do PRP and PRGF preparations to facilitate wound healing and tissue regeneration. Thus, we evaluated the levels of the selected major growth factors and pro-inflammatory cytokines in A-PRF and CGF extracts and compared the data with those of PRP and PRGF preparations. To reduce the individual-dependent differences in the growth factor levels, we collected sufficient volumes of peripheral blood samples from the same donors both in the presence or absence of anticoagulants and immediately prepared four types of platelet concentrates.

## Methods

### Preparation of PRP

Based on their characteristics and fractionation, the differences among PRP and PRP derivatives are concisely described in our previous article [1].

As previously described [5, 6], blood samples (11.5 mL) were collected using syringes or vacuum blood collection tubes equipped with 18G needles from seven non-smoking, healthy, middle-aged, male volunteers (37 to 68 years old) three times with a 2-week interval. Even though they are suffering from lifestyle-related diseases and receiving medication, these donors had no hindrance in daily life.

To quantify each blood cell component, peripheral blood (~12.0 mL) was collected using syringes containing acid citrate dextrose solution (ACD-A) (1.5 mL; Terumo, Tokyo, Japan). Because of its high efficiency, PRP was prepared using FDA-approved Ycellbio PRP preparation tubes (Ycellbio Medical Co., Ltd., Seoul, South Korea). As previously described [7], freshly collected blood samples were subjected to blood cell count and simultaneously transferred to funnel-shaped tubes and centrifuged at 3200 rpm (1800*g*) for 4 min (Table 1). After adjusting the level of the buffy coat, the tubes were centrifuged for an additional 4 min. The resulting PRP fractions (buffy coat) were collected using syringes equipped with long needles and stored at −80 °C until determination of growth factor levels and the in vitro bioassay using periosteal cells (*n* = 20). Small aliquots of the freshly prepared PRP were subjected to blood cell count.

**Table 1:** Centrifugation conditions for preparation of PRP, PRGF, A-PRF and CGF

Preparation type	Models	Rotor	Radius (mm)	Rotational speed		Time (min)
				rpm	*g*	
PRP	Kubota4000	swing	160	3,200	1,800	4 x 2
PRGF	Bti Endoret	swing	151.6	1,850	580	8
A-PRF	A-PRF	angle	105	1,300	198	8
CGF	Medifuge	angle	85	2,700 2,400 2,700 3,000	692 547 692 855	2 4 4 3

The study design and consent forms for all procedures performed with the study subjects were approved by the ethical committee for human subjects at Niigata University School of Medicine in accordance with the Helsinki Declaration of 1975 as revised in 2008.

### Preparation of PRGF

According to the manufacturer’s instructions, blood samples (~9.6 mL) were collected from the same volunteers using 18G needles and PRGF-Endoret^®^ tubes (BTI Biotechnology Institute, S.L., Miñano, Spain), which contained 0.2 mL sodium citrate. The tubes were centrifuged at 1850 rpm (580*g*) for 8 min (Table 1) [8]. Fraction 2 (a fraction above the buffy coat) was collected and stored at −80 °C until use. Small aliquots of the freshly prepared PRP were subjected to blood cell count.

### Preparation and homogenization of A-PRF and CGF

As described previously [7, 9], blood samples (~9.5 mL) collected without anticoagulants using vacuum plain glass tubes (A-PRF+: Jiangxi Fenglin Medical Technology Co. Ltd, Fengcheng, China) or conventional vacuum plain glass tube (Plain BD Vacutainer Tube; Becton, Dickinson and Company, Franklin Lakes, NJ, USA) from the same donors were immediately centrifuged by an A-PRF centrifugation system (A-PRF12: Dragon Laboratory Instruments Ltd., Beijing, China) or a Medifuge centrifugation system (Silfradent S. R. L., Santa Sofia, Italy) (for the conditions of centrifugation, see Table 1). After eliminating the red blood cell (RBC) fractions, the resulting A-PRF and CGF clots were placed on dry gauze to eliminate excess amounts of serum (~10 s) and then transferred to freezing tubes for determination of growth factor contents. Frozen samples stored at −80 °C were then minced, homogenized by disposable homogenizers (BioMasher II, Nippi, Inc., Tokyo, Japan), and centrifuged at 3000 rpm for 10 min at ambient temperature. The resulting supernatants were stored at −80 °C until use.

For determination of blood cell counts, another set of A-PRF/CGF clots was prepared from blood samples obtained from the same donors.

### Determination of blood cell counts

The numbers of blood cells were determined twice in the process of PRP preparation using an automated hematology analyzer (pocH-100iV diff; Sysmex, Kobe, Japan). First, RBCs, WBCs, and platelets were counted immediately after blood collection. Second, freshly prepared PRP and PRGF samples were directly submitted for the blood cell count. The obtained values were adjusted by their relative dilution factors.

As for A-PRF and CGF preparations, RBC, WBC, and platelet counts were determined by subtracting those counts in RBC fractions, supernatant acellular serum fractions, and A-PRF/CGF exudate fractions from those counts in whole blood samples, a method which we designated “indirect subtraction method.”

### Determination of growth factor and cytokine levels by ELISA

The concentrations of transforming growth factor-β1 (TGF-β1), platelet-derived growth factor-BB (PDGF-BB), and vascular endothelial growth factor (VEGF) in frozen PRP, PRGF, A-PRF, and CGF samples were determined using human TGF-β1, PDGF-BB, and VEGF Quantikine ELISA kits (R&D Systems, Inc., Minneapolis, MN, USA). Concentrations of interleukin-1β (IL-1β) and interleukin-6 (IL-6) were determined using the IL-1β human ELISA kit and IL-6 high sensitivity human ELISA kit (Abcam, Cambridge, MA, USA).

### Evaluation of cell proliferation

Because alveolar periosteum is closely contributed to periodontal skeletal tissue regeneration, we used human alveolar bone-derived periosteal cells for evaluation of efficacy of the PRP derivatives. The periosteal cells were obtained and expanded as described below. With informed consent, human periosteum tissue segments were aseptically dissected from the periodontal tissues of the healthy buccal side of the retromolar region of the mandibles of non-smoking volunteers [10]. Small periosteum pieces were expanded to form cell-multilayered periosteal sheets (φ30–40 mm) in humidified 5 % CO_2_, 95 % air at 37 °C with medium 199 (Invitrogen, Carlsbad, CA, USA) supplemented with 10 % fetal bovine serum (FBS) (Invitrogen), 25 μg/mL ascorbic acid 2-phosphate, and antibiotics. Then, the periosteal sheets were enzymatically digested to release single cells. The resulting cells were further expanded in DMEM supplemented with 10 % FBS.

Single cells were seeded at a density of 1 × 10^4^ in 6-well plates and pre-cultured for 24 h in 1 % FBS-containing DMEM. The medium was replaced with the same fresh medium containing PRP, PRGF, A-PRF extract (A-PRF*ext*) or CGF extract (CGF*ext*) (0.625, 1.25, 2.5, 5.0, 10 %), and the cells were further incubated for 48 h. Because embedded in fibrin gel by the treatment of PRP or PRGF, cells were not enzymatically harvested for determining cell counts. Instead, cells were photographed and counted in three randomly selected views using Image-PRO Plus software (Media Cybernetics Manufacturing, Warrendale, PA, USA) [11] (*n* = 4). Although cells were not embedded in the medium containing A-PRF*ext* or CGF*ext*, to compare these efficacies with those of PRP or PRGF, cell proliferation was evaluated by the same method.

### Statistical analysis

The data were reported as the mean value ± standard deviation (SD). For multi-group comparisons, statistical analyses were performed to compare the mean values using one**-**way analysis of variance (ANOVA) followed by Dunn’s or Tukey’s multiple comparison test (SigmaPlot 12.5; Systat Software, Inc., San Jose, CA, USA). *P* values <0.05 were considered significant.

## Results

**Fig. 1:**
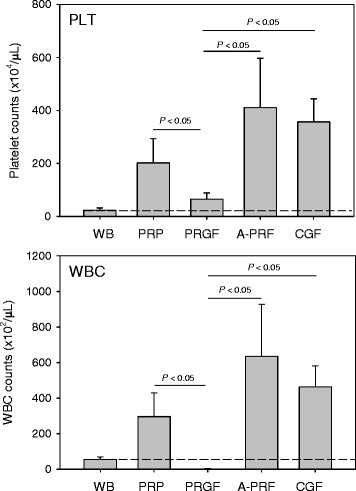
Comparisons of platelet counts in whole blood (WB) samples and PRP and PRGF, A-PRF and CGF preparations (*n* = 12 (A-PRF, CGF) or 20 (PRP, PRGF))

Numbers of platelets in PRP and PRGF preparations are shown in Fig. 1 (upper panel). Platelets were significantly concentrated both in the PRP and PRGF preparations, and the concentration rate of PRP preparations was substantially higher than that of PRGF preparations (8.79-fold vs. 2.84-fold). Numbers of platelets in A-PRF and CGF preparations calculated by the indirect subtraction method are also shown in Fig. 1 (upper panel). Platelets were significantly concentrated also in both A-PRF and CGF preparations with the concentration rates of 17.85-fold and 15.51-fold, respectively.

Numbers of WBCs in PRP and other PRP derivatives are shown in Fig. 1 (lower panel). WBCs were similarly concentrated in these platelet-concentrated preparations (PRP: 5.51-fold, A-PRF: 11.87-fold, CGF: 8.63-fold). However, only an exception was PRGF preparations; WBCs were almost completely eliminated from PRGF preparations (0.015-fold).

**Fig. 2:**
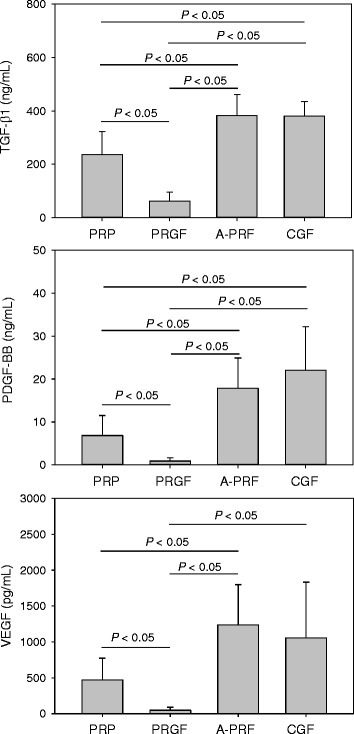
Concentrations of growth factors (TGF-β1, PDGF-BB, VEGF) in PRP, PRGF, A-PRF, and CGF preparations (*n* = 20)

The concentrations of growth factors in PRP, PRGF, A-PRF, and CGF preparations are shown in Fig. 2. The order of growth factor levels (TGF-β1, PDGF-BB, VEGF) were A-PRF ≥ CGF > PRP >> PRGF. PRGF preparations contained the lowest amounts of growth factors.

**Fig. 3:**
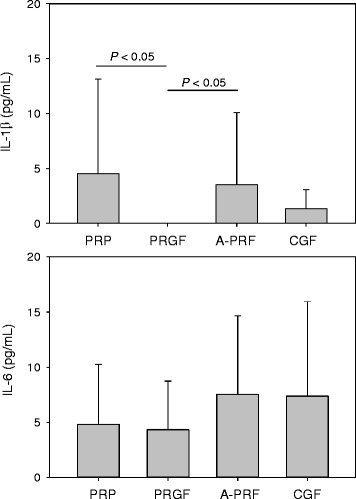
Concentrations of the pro-inflammatory cytokines (IL-1β, IL-6) in PRP, PRGF, A-PRF, and CGF preparations (*n* = 20)

The concentrations of inflammatory cytokines in PRP, PRGF, A-PRF, and CGF preparations are shown in Fig. 3. IL-1β in PRGF preparations was under detectable levels, while it showed a similar level in other three preparations. For IL-6, in contrast, there were no significant differences among these preparations.

**Fig. 4:**
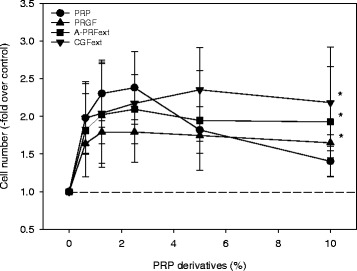
Effects of PRP, PRGF, A-PRF, and CGF on the proliferation of human periosteal cells. Cells were treated with PRP preparations, PRGF preparations, A-PRF extracts, or CGF extracts at the indicated doses for 48 h in 1 % FBS-containing medium. *P < 0.05 compared with the controls without nay addition (*n* = 4)

The effects of individual PRP derivatives on the proliferation of human periosteal cells are shown in Fig. 4. As shown in the preceding study [12], PRP preparations exerted a biophasic effect with the maximal effects observed at 2.5 %, while in PRGF preparations, A-PRF*ext* and CGF*ext* stimulated cell proliferation in a dose-dependent manner (0.625–10 %). The apparent order of potency was PRP > CGF > A-PRF > PRGF.

## Discussion

Although the growth factor contents in PRF and CGF preparations and their bioactivities have been demonstrated in in vitro studies by several independent groups [8–11, 13–20], many clinicians still believe that the regenerative effects of PRF/CGF are solely due to fibrin clots. We speculate that this discrepancy may be caused by two major factors. First, the initial report on PRF by Choukroun and his co-workers showed that PDGF-BB, TGF-β1, or IGF-I is not significantly concentrated in PRF preparations [21]. Second, the preparation protocols of PRF extraction are not fully disclosed in several articles and likely varied with the individual groups. In the previous study [7], we demonstrated that intense compression of PRF preparations, which is designated as CGF preparations in this study, with dry gauze fully removes PRF exudate and substantially reduces the content of growth factors. Therefore, we concluded that the major source of growth factors in PRF preparations is its exudate; however, as a minor source, growth factors are thought to be secured by fibrin fibers.

To confirm these observations, we recently examined the angiogenic activity of PRF/CGF preparations in endothelial cell cultures and the chick embryo chorioallantoic membrane (CAM) assay [22]. As a result, it was demonstrated that PRF/CGF preparations are somewhat more potent in angiogenesis than PRP preparations. To further assure the growth factor contents in the self-clotted PRP derivatives, in this study, we compared the growth factor contents in four types of PRP derivatives (PRP, PRGF, A-PRF, CGF) prepared from the same donors. The main finding of this study was that both A-PRF and CGF preparations contained TGF-β1, PDGF-BB, VEGF, IL-1β, and IL-6 at levels similar to or higher than PRP preparations. The expected proliferative effects of both A-PRF and CGF extracts were demonstrated in the in vitro assay using human periosteal cells, which give rise to osteoblasts involved in periodontal skeletal regeneration. Therefore, as do PRP preparations, these self-clotted PRP derivatives are expected to function not only as a scaffolding material but also as a reservoir to deliver certain growth factors and pro-inflammatory cytokines at the implantation sites.

In the previous study [12], we found that PRP and A-PRF preparations exert distinguishable actions on periosteal cell proliferation. Because both IL-1β and IL-6 are known to be produced by WBCs [23], and because WBCs are not included in PRGF preparations, we thought that the bi-phasic effects of PRP preparations may be attributed to WBCs. Furthermore, if WBCs are highly concentrated in A-PRF, it is expected that IL-1β and IL-6 are concentrated at higher levels to exert negative effects at higher doses of A-PRF extracts in this study. As Choukroun intended [3], we observed that WBCs, as well as platelets, were highly concentrated in A-PRF preparations. Similarly, WBCs were found to be concentrated in CGF preparations. In addition, the inflammatory cytokines were not exceptionally concentrated at higher levels in PRP preparations, and no strong positive correlation between WBC counts and pro-inflammatory cytokine levels was observed in PRP preparations (data not shown). Therefore, we speculate at present that the negative effects of PRP preparations at higher doses may not be due to these pro-inflammatory cytokines or WBCs. However, at the same time, we are concerned how accurately the indirect subtraction method determines WBC and platelet (PLT) counts, because this method does not count any possible adhesion-dependent loss of blood cells or nonuniformity of cell distributions especially in red thrombus. Thus, further studies are needed to perform to obtain convincing evidence to explain this discrepancy.

## Conclusions

The present study clearly demonstrated that both A-PRF and CGF preparations contained significant amounts of growth factors, which makes us to believe that A-PRF and CGF preparations would not only function as a scaffolding material but also as a reservoir to deliver certain growth factors at the site of application. Accordingly, it is expected that these two preparations are more potently capable of inducing angiogenesis and subsequent wound healing/tissue regeneration than PRP preparations.

## Abbreviations

ACD: Acid citrate dextrose solution
ANOVA: Analysis of variance
A-PRF: Advanced platelet-rich fibrin
A-PRF*ext*: A-PRF extract
CGF: Concentrated growth factors
CGF*ext*: CGF extract
ELISA: Enzyme-linked immunosorbent assay
IL-1β: Interleukin-1β
IL-6: Interleukin-6
PDGF-BB: Platelet-derived growth factor-BB
PLT: Platelet
PRGF: Plasma rich in growth factors
PRP: Platelet-rich plasma
RBC: Red blood cell
TGF-β1: Transforming growth factor-β1
VEGF: Vascular endothelial growth factor
WBC: White blood cell
